# A summary of the main themes and findings presented at the ASM Intermountain Branch meeting (2024)

**DOI:** 10.1128/msphere.00481-24

**Published:** 2024-07-09

**Authors:** Jay Radke, Javier Ochoa-Repåraz, Jamee Nixon, Sajal Acharya, Haley Bridgewater, Joshua Burger, Abigail Cheever, Robert Darby, William Doyle, Alka Gaur, Eva Githuku, Rose Goodman, Topher Haynie, Hannah Hedelius, Kristina Hill, Misha Iqbal, Salma Laabi, Carlos Moreno, Melinda Moss, Nagama Parveen, Naomi Rapier-Sharman, Sara Sadeghi, Saeed Saleh, Sean Schumacher, Miranda Sharp, Noah Souza, Soni Thapa, Shule Aggabao, David Amsbury, Sheena Isabelle Bautista, Atalie Bogh, Aaron Bohn, Cade Brink, B. Shaun Bryner, James Cannon, Scot Carrington, Hayzen Chamberlain, Alex Cherry, McKaylin Cole, Edgar Corrales, Caz Cullimore, Sophie Daines, Payson Danielson, Monterey Domike, Matthew East, Bronwyn Ellis, Taryn Evans, Zach Fears, Paige Fellars, Tate Fisher, Braxton Floyd, Trenton Gibson, Mason Gueller, Heather Gupta, Jacob Gwilliam, Mackenzie Hansen, Jacob Hardy, Christopher Harrell, Rebecca Hassell, Wesley Hendricks, Colby Hendrix, Hirsche Henstrom, Kelly Hernandez Sanguino, George Higgins, Hyunbi Hwang, Matt Jackson, Conner Jensen, Austin Johnson, Chloe Kang, Sehi Kim, Alexandra LaFollette, Phoenix Larsen, Abbey Larson, Bryson Leary, Jayden Longhurst, Michael Mann, Isreal Martinez, Brooklyn Matthews, Cody McStraw, Ninahazwe Mireill, Rachel Moffat, Peter Mourik, Madelyn Mudrow, Mailon Odell, Blake Oler, Natalie Olsen, Nazanin Paymard, Spencer T. Payne, Levi Pearson, Josh Peter, Tiffani Peterson, Daniel Puentes Navarro, Kyla Radke, Joseph Richardson, Russell Ridd, Akir Rowe, Rylan Schmanski, Jacob Scott, Samuel Scott, Mya Simpkins, Madalyne Sisk, Tyler Smith, Brinley Smith, Jacob Sy, Gisselle Trejo, Bartel Van Oostendorp, Ethan Walbom, Rebecca Whetten, Dallin Zollinger, Miriam Braunstein, Donald P. Breakwell, Anirban Chakraborty, Matthew Crook, Michele Culumber, Wayne Hatch, Victor M. Jimenez, Jr., Wales P. Nematollahi, Michael Olson, Mark Poritz, Seth Ririe, Jeffrey Schachterle, Lisa Wiltbank, Todd Kelson, Brett E. Pickett

**Affiliations:** 1 Biological Sciences, Boise State University, Boise, Idaho, USA; 2 Boise Veterans Administration Medical Center, Boise, Idaho, USA; 3 Idaho Veterans Research and Education Foundation, Boise, Idaho, USA; 4 Biology, Northwest Nazarene University, Nampa, Idaho, USA; 5 Biological Sciences, Idaho State University, Pocatello, Idaho, USA; 6 Microbiology and Molecular Biology, Brigham Young University, Provo, Utah, USA; 7 Microbiology and Immunology, Noorda College of Osteopathic Medicine, Provo, Utah, USA; 8 Biology, Utah State University Eastern, Price, Utah, USA; 9 Microbiology, Weber State University, Ogden, Utah, USA; 10 Biology, Utah Valley University, Orem, Utah, USA; 11 Biology, Brigham Young University Idaho, Rexburg, Idaho, USA; 12 Medicine, Huntsman Cancer Institute, Salt Lake City, Utah, USA; 13 Microbiology, Immunology, and Pathology, Colorado State University, Fort Collins, Colorado, USA; 14 Semi-Retired Microbiologist, Salt Lake City, Utah, USA; 15 Biological Sciences, Snow College, Ephraim, Utah, USA; 16 Faro Molecular, Salt Lake City, Utah, USA; University of Michigan, Ann Arbor, Michigan, USA

**Keywords:** branch meeting, microbiology, phages, environmental biology, infectious diseases, clinical microbiology, applied microbiology, cellular biology, molecular biology

## Abstract

The annual meeting for the Intermountain Branch was held in April 2024 on the campus of Brigham Young University. There were 127 branch members from Utah, Idaho, and Nevada who attended the meeting and were composed of undergraduate students, graduate or medical students, and faculty. This report highlights the diversity of, and the emerging trends in, the research conducted by American Society for Microbiology members in the Intermountain Branch.

## MEETING SUMMARY INFORMATION

**Fig 1:**
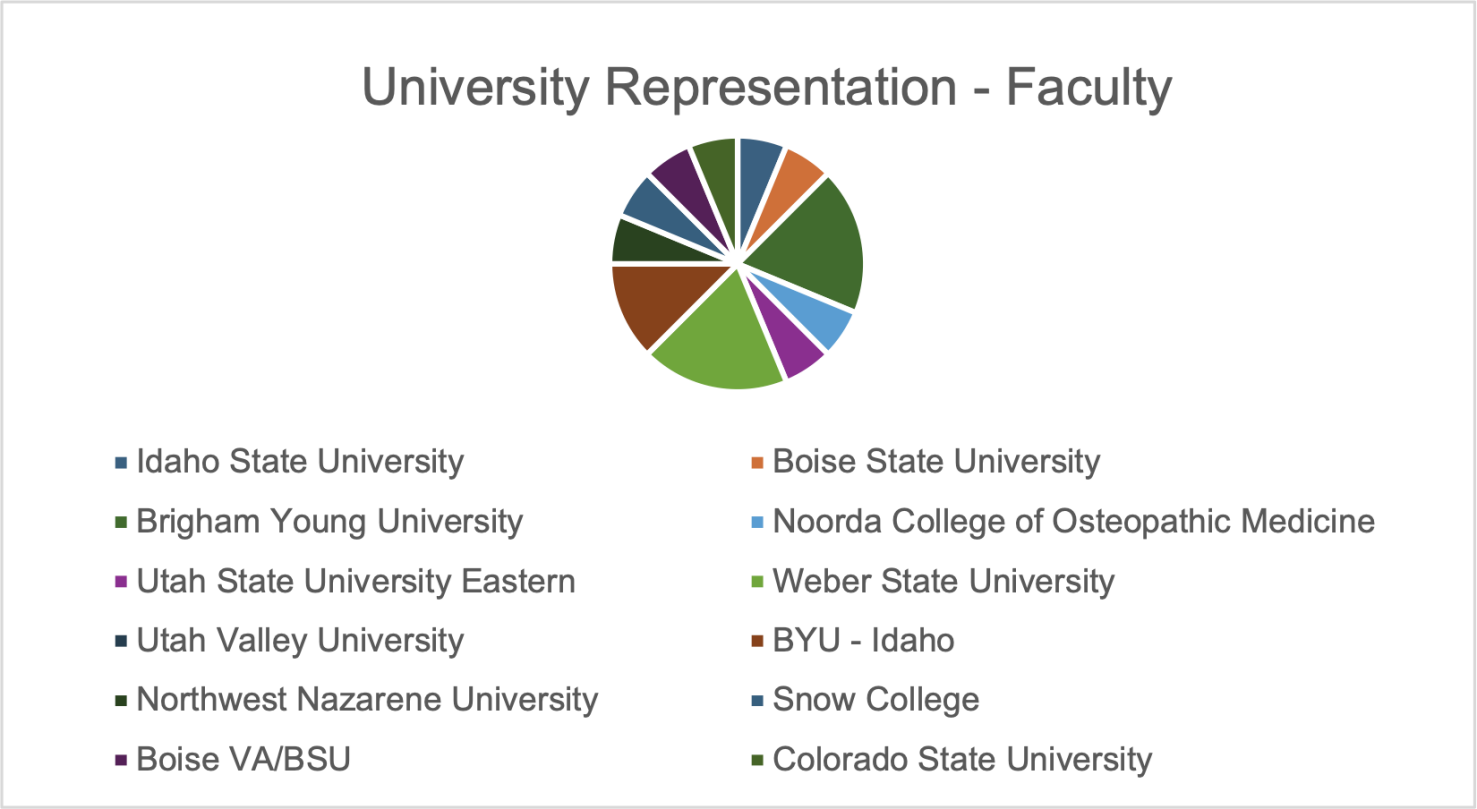
A pie chart showing the distribution of faculty participants, representing 12 different institutions in the Intermountain region.

**Fig 2:**
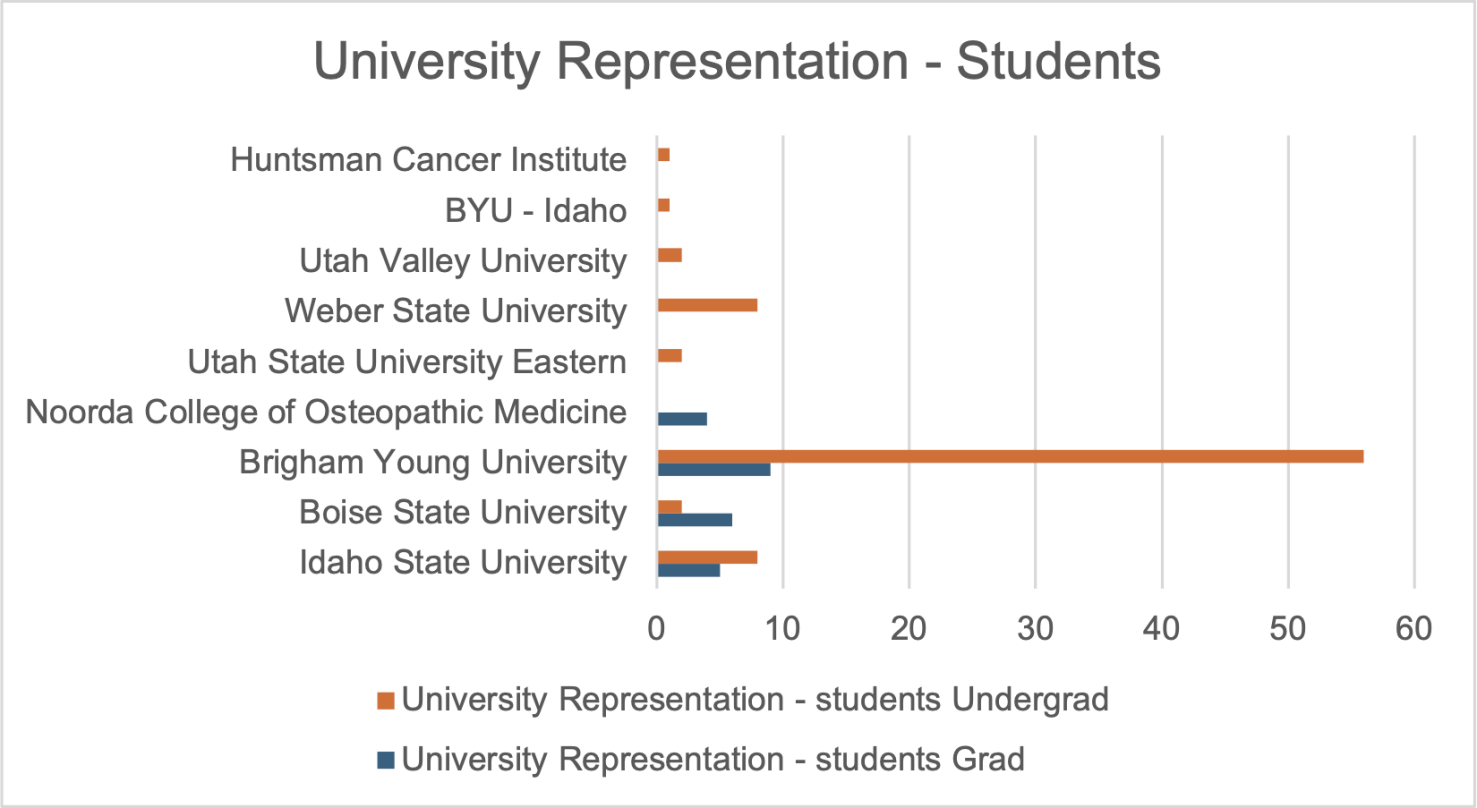
A bar chart showing the numbers of student participants by institution and a higher overall number of undergraduates than graduate students participating in the meeting.

**Fig 3:**
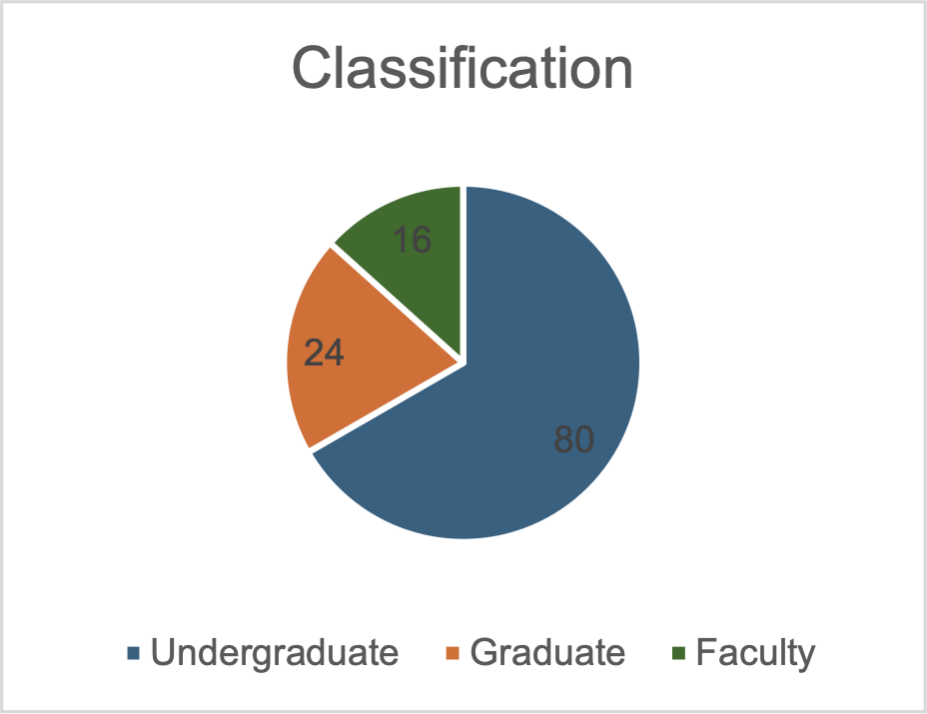
A pie chart showing the proportions and numbers of attendees from each of three categories—undergraduate, graduate, or faculty.

**Fig 4:**
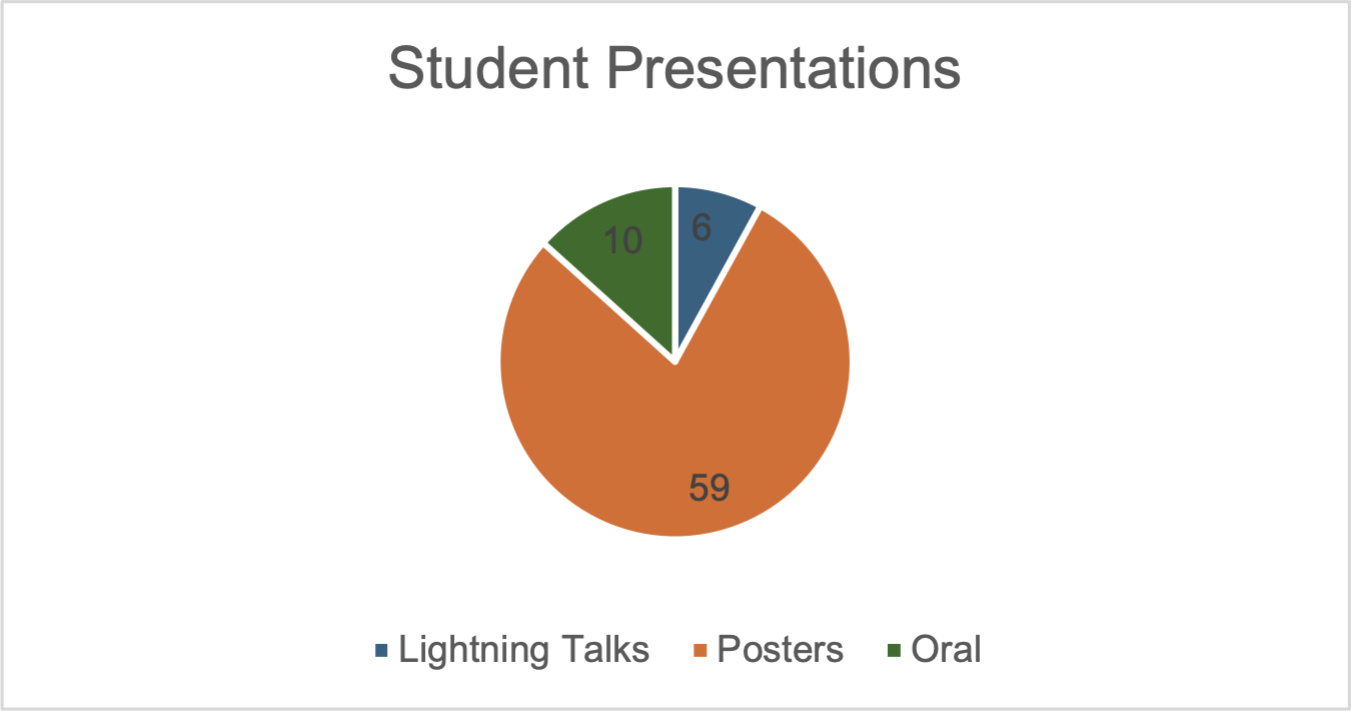
A pie chart displaying the three categories of presentations given by the 75 presenters at the conference, including 10 oral, 59 posters, and 6 lightning presentations.

The annual meeting for the Intermountain Branch of the American Society for Microbiology (ASM) was held in April 2024 on the Brigham Young University campus in Provo, UT. The branch includes members across all of Utah as well as parts of both Idaho and Nevada. Representation from different institutions was more diverse with faculty than with student participation (Fig. 1). Although the majority of trainees in attendance were undergraduates (86; 68%), a substantial number of attendees were graduate or medical students (24; 19%) and faculty (17; 13%) (Fig. 2 and 3). Presenters from nine distinct undergraduate, professional, and/or research institutions within the branch boundaries contributed abstracts to the meeting, including Boise State University, Brigham Young University, Brigham Young University Idaho, Huntsman Cancer Institute, Idaho State University, Noorda College of Osteopathic Medicine, Utah State University Eastern, Utah Valley University, and Weber State University. The format of the presentations was oral, poster, or 3-minute lightning talks (Fig. 4).

### Keynote address

The keynote speaker for the meeting was Miriam Braunstein, PhD, which was made possible as part of the ASM Distinguished Lecture series. The keynote address communicated recent increases in the number of infections with non-tuberculous mycobacteria (NTM) and past work by the community to develop phage-based therapeutics for compassionate use in the clinic. Recent results from Dr. Braunstein’s lab to better characterize therapeutic phages that are capable of targeting NTMs such as *Mycobacterium abscessus* were presented. Dr. Braunstein presented data addressing the potential of phages to enter and act in the intracellular environment in which NTM bacteria reside. Differences between the intracellular activity of individual phages were uncovered, which will require additional research to better understand.

## PRESENTATION THEMES

Given the history of presentations in the branch, we grouped the presentations into three of the scientific units that are emphasized by ASM, including Applied and Environmental Microbiology, Cellular and Molecular Microbiology, as well as Clinical and Public Health Microbiology.

### Applied and Environmental Microbiology

Students presented data collected from experiments with samples obtained from a heterogeneous pool of environments, such as watersheds, soils, and plants from the Idaho-Nevada-Utah region and ocean hydrocarbon seeps. Analyses included bacterial and bacteriophage populations for multiple basic research and agricultural applications. The high-level categories of presentation topics in this theme included examining the quantity and diversity of microbes in aquatic bodies, manipulating and/or improving the ability of microbes to perform various processes, identifying microbial-based mechanisms to reduce the effect of pathogenic bacteria in various systems, as well as surveying and characterizing the diversity of bacteria or phages in specific environments. We believe that continued research in applied and environmental microbiology at institutions in the Intermountain Branch region will provide a more in-depth understanding of the diversity and capabilities of microbes and how they can be used to benefit other systems.

One of the oral presentations within this theme focused on the ecological networks, genomic novelties, and potential bioremediation of microbes from polychlorinated biphenyls (PCB)-contaminated environments. Briefly, a microcosm was constructed from contaminated mud from the northeastern United States, as well as filtered pond water under aerobic or anaerobic conditions prior to plating an inoculum from the microcosm onto agar plates. Approximately 30 PCB-resistant bacterial colonies grew on the plate and included members of the following genera: *Paenibacillus*, *Clostridium*, *Rhizobium*, *Methylversatalis*, and *Sphingobacteria*. Further analysis should enable the identification of species, characterization of the metabolic diversity, and characterization of the PCB-tolerant bacteria from this microcosm environment.

A poster presentation focused on constructing a four-enzyme synthetic pathway in a single reaction that is capable of generating rare sugars such as D-tagatose and D-allulose from lactose-heavy dairy streams. A single-pot reaction yielded these rare sugars with concentrations in the mg/mL range, with significantly higher amounts of these rare sugars being produced when higher levels of lactose were present.

### Cellular and Molecular Biology (includes microbial physiology and metabolism, structural and cellular microbiology, host-microbe interactions, microbial genetics and genomics, computational modeling, and microbial evolution)

The Cellular and Molecular Biology section included the largest percentage of posters presented. The works included a broad array of disciplines that included microbiology and pathogenesis, as well as cellular and molecular biology aspects of the host, immunology, and/or molecular genetics. Some of the research topics included were in the areas of autoimmunity and neuroimmunology, such as multiple sclerosis, as well as cancer and infectious diseases, among others. The main subtopics that emerged in this session, which contained the most presentations, dealt with characterization and annotation of new phages, effects of epigenetics in eukaryotes, modulating the eukaryotic immune response using chemicals or cells, characterization and manipulation of prokaryotic nucleic acids, characterizing mechanisms of pathogenesis or other prokaryotic gene products, and applying computational analysis methods. We believe that continued research in cellular and molecular biology at Intermountain Branch institutions will improve our mechanistic knowledge of how microbes cause disease and how they can be manipulated to improve the world around us.

An oral presentation relating to this theme involved identifying novel therapeutic targets for alcohol use disorder (AUD). In AUD, ethanol increases levels of blood dopamine that binds to dopamine D2 receptors and alters extracellular cytokine levels. An upstream cytokine signaling event is likely involved since dopamine neurons are insensitive to ethanol and dopamine cannot cross the blood-brain barrier. Multiplexed cytokine experiments are underway to characterize any differences that exist in untreated and ethanol-treated cells. Elucidating the pathway(s) of peripheral ethanol on neuronal dopamine production, including specific cell types and cytokines, can increase our understanding of AUD and can potentially contribute to future immunotherapeutic treatments for AUD.

Another presentation in this session reported the implementation of a computational approach to better understand the immunological similarities and differences between contrasting diseases in B cells (lymphoma and systemic lupus erythematosus). The results from this analytical workflow could delineate new mechanisms that can be leveraged to treat these B-cell diseases. Briefly, the input for this approach uses differentially expressed genes from disease vs normal samples collected from human patients. These data are then used to identify gene ontology terms, protein-protein interactions, and transcriptional mechanistic markers for each disease. A novel scoring metric is incorporated into the algorithm to weigh the perceived role of each result. The results from this novel tool should contribute to the continued development of immune-focused treatment options for B-cell lymphomas and other cancers.

### Clinical and Public Health Microbiology

As in the Cellular and Molecular Biology section, works presented in the Clinical and Public Health Microbiology section also included various topics and experimental tools. The presentations that were relevant to this theme could be categorized into subtopics that included identifying, manipulating, or characterizing therapeutic and/or diagnostic methods for cancer, autoimmune, and neurological diseases; characterizing microbes and their pathogenic gene products; and quantifying the production or effect of phages or antimicrobial compounds. Bioinformatics tools and results were also presented, using samples from various origins, including gut microbiota, animal models, and humans. We believe that continued research in the areas of clinical and public health microbiology by members of the Intermountain Branch will improve our understanding of infectious diseases and how to design biological systems to improve human health.

One of the oral presentations within this theme evaluated the application of Chimeric antigen receptor (CAR) T cells to treat Graves’ disease (GD), which is an autoimmune disorder that results from autoreactive B cells against thyroid-stimulating hormone receptor (TSHR) and results in the overproduction of thyroid hormones. TSHR epitopes that are most compatible with CAR T cells and most likely to interact with B cells in GD patients were selected. These CAR T cells showed significant binding to anti-TSHR antibodies and activation of T-cell cytotoxicity. An *in vitro* assay showed that when engineered GD autoreactive B cells are co-incubated with the CAR T cells, the latter are activated and kill the autoreactive B cells. Impressively, the CAR T cells do not bind to or eliminate healthy B cells, making this a safe treatment with minimal side effects; however, further work is needed to validate this potential treatment for other autoimmune diseases.

A separate oral presentation in this session evaluated the potential immunomodulatory role of psilocybin, which is generally used to treat depression and substance abuse. In particular, the study quantified the cytotoxic and immunomodulatory effects of psilocybin and psilocin, which were administered at different doses, on both resting and lipopolysaccharide-activated murine macrophages. The study showed that psilocybin is almost twice as cytotoxic as psilocin, while resting macrophages had a significantly greater release of TNF-α, with lower treatment doses having a larger effect on cytokine levels than higher doses. Psilocin, but not psilocybin, induced a significant increase in IL-10 post-treatment, leading to the conclusion that psilocin, but not psilocybin, exerts anti-inflammatory effects on classically activated macrophages.

## CONCLUSIONS

The ASM Intermountain Branch continues to have strong participation from both established members and student researchers from institutions in our region. We anticipate that the major themes presented in this conference at least partially represent those that are common in other branch meetings and reflect the importance of research by members of the branch and of the national organization pertaining to the three scientific units described above. We expect that continued effort in these primary areas of research will enable improved mechanistic understanding of pathogens, their hosts, and how to prevent various diseases.

